# Supplementary material for: Experiences and care trajectories of persons living with Hepatitis B in Senegal: A qualitative study
Source: PLOS Glob Public Health. 2025 Nov 7;5(11):e0005376. doi: 10.1371/journal.pgph.0005376 (PMC12594332; doi:10.1371/journal.pgph.0005376)

Inclusivity in global research

PLOS’ policy on inclusivity in global research aims to improve transparency in the reporting of research performed outside of researchers’ own country or community and ensures that PLOS publications reporting global research adhere to high standards for research ethics and authorship. Authors of relevant research articles may be asked to complete the questionnaire below, which outlines ethical, cultural, and scientific considerations specific to inclusivity in global research. This questionnaire may be requested when researchers have travelled to a different country to conduct research, if research uses samples collected in another country, research with Indigenous populations or their lands, or if research is on cultural artefacts. Researchers travelling to another country solely to use laboratory equipment will not normally be required to complete the questionnaire. However, the questionnaire can be requested at the journal’s discretion for any submission – if you have been requested to complete this questionnaire by the PLOS journal you submitted to, please do so.

Please complete the questionnaire below and include this as a Supporting Information file with your manuscript. Note that if your paper is accepted for publication, this checklist will be published with your article in the supporting information files. Please ensure that you reference the checklist in the main body of your manuscript. We suggest adding a subsection ‘Inclusivity in global research’ to your Methods section and adding the following sentence: “Additional information regarding the ethical, cultural, and scientific considerations specific to inclusivity in global research is included in the Supporting Information (SX Checklist)”

The questions have been designed to be applicable to a wide range of study types, and there are subsections for both human subjects research and non-human subjects research. If any of the questions are not relevant to your research please mark them as “N/A” as appropriate.

**Ethical considerations, permits and authorship**

*This section is applicable to all research types.*

Provide details as to who granted permissions and/or consent for the study to take place in the Methods section of your manuscript. This should include the names of **all** ethics boards, governmental organizations, community leaders or other bodies that provided approval for the study. If individuals provided approval, refer to these people by their role or title but do not list their name(s).

| Reported in the “Material and Methods” section (page 5- 7) |
| --- |

If there were any deviations from the study protocol after approval was obtained please provide details of these changes in the Methods section of your manuscript.

Did this study involve local collaborators that are residents of the country where the research was conducted or members of the community studied? If you do not have any authors from said communities, please provide an explanation for this below.

| Reported on page 1 - 6 - 26 |
| --- |

Everyone listed as an author should meet PLOS’ criteria for authorship and all individuals who meet these criteria should be included in the author bernal's Authorship Policy.

**Human subjects research (e.g. health research, medical research, cross-cultural psychology)**

Did you obtain written informed consent from a representative of the local community or region before the research took place? How did you establish who speaks for the community? Details of written informed consent obtained from study participants should be reported separately in the Methods section of your manuscript.

| Reported in second/fourth paragraph of the “ Material and Methods” section (page 5-6) and ethical considerations (page 7)  The study was hospital-based and followed a group of chronic hepatitis B patients. To recruit participants, we worked closely with physicians and cohort project teams who facilitated contact between researchers and eligible patients. Because the study was hospital-based and focused on a population already enrolled in a care protocol, written informed consent was not required from local or regional community representatives. However, verbal and written consent was obtained from each participant prior to inclusion in the cohort. Verbal consent was obtained prior to participation in this study. All participants received detailed information about the study objectives, methods, and use of results, and their participation was completely voluntary. |
| --- |

How did members of the local community provide input on the aims of the research investigation, its methodology, and its anticipated outcome(s)?

When engaging with the local community, how did you ensure that the informed consent documents and other materials could be understood by local stakeholders?

Will the findings of the research be made available in an understandable format to stakeholders in the community where the study was conducted (e.g. via a presentation, summary report, copies of publications, etc.)? Please provide details of how this will be achieved.

| Reported in the section “Material and Methods” section (page 5-6-7)  **1. Comprehensibility of documents and consent forms**  Although we did not obtain consent from a local representative, the aims and conditions of participation in the study were explained to participants in the local language, Wolof, to ensure clear understanding. This was to ensure that all participants fully understood the implications of their participation and felt comfortable asking questions or seeking clarification.  **2. Sharing findings with local stakeholders**  The results of this research have already been shared with various stakeholders  - Restitution Workshop: The results were presented at a workshop attended by national and regional stakeholders and people living with hepatitis B in January 2024  - Scientific days: The data were disseminated as posters at the AIDS Scientific Days in Senegal in 2022.  - International Hepatitis Day: The results were also shared at the World Hepatitis Day in July 2023.  **3. Plans for future dissemination of results**  We plan to produce clear and accessible fact sheets that will be distributed to all people followed in the cohort. These sheets will summarize the key findings and recommendations of the study in an understandable and adapted format.  4. **Communication impact of the study**  As a result of the study, physicians involved in the follow-up of the cohort have intensified their communication with patients, incorporating clearer and more appropriate information about hepatitis B. |
| --- |

**Non-human subjects research using specimens/ animals collected as part of the study, or those housed in archival collections. Examples include archaeology, paleontology, botany and zoology.**

Did the permission you obtained from a local authority to perform the study include an agreement on access to outputs and benefit sharing? This may include procedures to enable fair distribution of the benefits and resources arising from the research performed. Please include any details of Prior Informed Consent and Benefit Sharing Agreements obtained. These may be required by field-specific regulations, for example the Convention on Biological Diversity (CBD) and the associated Nagoya Protocol.



If the material used in your study was imported, please A) provide the year it was imported and B) indicate whether permits were obtained to import/export the materials used, C) provide details of any permits obtained. If this information is not available, please indicate this.



If you used archival specimens, please state how the material used in your study was acquired by the institute it is held in and provide details of any permits obtained for the original excavations/ sample collection. If this information is not available, please indicate this.



How was the potential cultural significance of the materials collected in your study to local communities considered in your research design? Were Indigenous peoples and/or local researchers and institutions involved with archaeological excavations / collection of specimens? If so, please provide a description of their involvement.



If your manuscript includes photographs of human remains please indicate whether authors obtained permission from descendants or affiliated cultural communities to do so.


Supplement: S1 Checklist — (DOCX) [file pgph.0005376.s001.docx]
