# Supplementary material for: Experiences and care trajectories of persons living with Hepatitis B in Senegal: A qualitative study
Source: PLOS Glob Public Health. 2025 Nov 7;5(11):e0005376. doi: 10.1371/journal.pgph.0005376 (PMC12594332; doi:10.1371/journal.pgph.0005376)
Supplement: S1 File — (PDF) [file pgph.0005376.s002.pdf]

Semi structured Interview Guide

Introduction and consent

Objective

This study aims to explore the experiences of individuals with hepatitis B in the SEN-B cohort, focusing on their journey from initial diagnosis to their care pathway. This includes an examination of their treatment choices, access to healthcare, and social support systems. Your input will contribute to enhancing the care for people with hepatitis B in Senegal.

Study Procedure and Participant Consent

This interview will explore your personal experiences living with hepatitis B, including your care and treatment choices and any challenges encountered. With your permission, the discussion will be audio-recorded to ensure accuracy, and all information will remain strictly confidential. Participation is entirely voluntary; you may withdraw at any time without impacting your medical care. Your identity will be protected through pseudonyms in all study documents, and only authorized research team members will have access to the data. Do you consent to participate in this study?

Sociodemographic characteristics

Date of the interview	
Time Start - Time End	
Patient ID	
Age	
Sex	
Place of residence	
Marital status	
Number of children	
Employment	
Date of HBV diagnosis reported	

Section A: perceptions and knowledge about Hepatitis B

1. Have you ever heard of the term "hepatitis B" ?
2. What comes to mind when you hear the words "hepatitis B"?
3. What term do you use to name hepatitis B in Wolof? (probe about A, B, C)
4. In your opinion, what does the "B" in hepatitis B refer to?
5. What questions do you have about your illness?
6. In your opinion, what causes this disease?
7. In your opinion, what are the signs of this disease? (probe about absence of signs/meaning)
8. In your opinion, how does this disease progress? (probe about complications)
9. In your opinion, which groups are most at risk? (by age, sex, lifestyle, behavior)
10. In your opinion, how can one protect themselves from hepatitis B?
11. Is this an epidemic or a disease? (ancient vs. new / cyclical vs. permanent)
12. What treatments are available for HBV, in your opinion? (probe about biomedicine, traditional)
13. Do you believe that a person with hepatitis B can be cured? Why or how do you think that is possible?
14. What rumors have you heard about hepatitis B?

Section B: diagnosis experience

15. What illness did you think you had before learning your HBV+ serological status?
16. Tell us how you learned about your HBV infection. (probe: what year, circumstances)
17. For what reasons did you decide to get tested at that particular time and not before?
18. Who informed you of your status? What did they tell you? (describe terms used)
19. How did you react following the announcement?
20. What questions did you ask the person who told you your status?
21. How do you evaluate the way your status was communicated to you?
22. What is your assessment of hepatitis B screening? (probe: cost, location, infrastructure)
23. How can these constraints be managed in your opinion?

Section C: disclosure and stigma

24. Did you share your status after being informed? If yes, with whom?
25. How did they react?
26. What prompted you to share your status?
27. Did you encourage those close to you to get tested?
28. Where did your family get tested? How did they proceed? *(*Probe: all together? one by one? same day?)
29. What experiences of stigma related to your illness have you faced?

Section D : attitudes, community life, and management of transmission risk

30. Since discovering your status, have you changed your attitude toward your loved ones?
31. How do you manage the risk of transmission within your family? Consider the following aspects: sexual practices, dietary habits, and the sharing of tools.
32. Have you discussed about hepatitis B with other people?
33. Do you have ancestors or relatives who have been affected by it? Tell us about it.
34. What is the most memorable event related to your illness that has had a lasting impact on you?

Section E : therapeutic itineraries

35. What was your first therapeutic recourse?
36. Where did you go for your first treatment recourse?
37. How did you secure funding to finance this treatment??
38. Who assisted you with this treatment process? Please share your experience with the treatment.
39. What is your assessment of this recourse?
40. Where did you go for your second therapeutic pathway?
41. Where did you go for your third therapeutic pathway?
42. What do you think are the barriers to accessing care after learning about your status?
43. What solutions do you think could help overcome these constraints?
44. According to doctors, hepatitis B is a chronic disease, and its treatment can last a lifetime. What are your thoughts on this?

Section F : information seeking

45. From which sources do you typically obtain information regarding your concerns?
46. What do you believe should be explained to individuals affected by hepatitis, like yourself?

We are at the end of our interview. Would you like to add anything?
