# Supplementary material for: Experiences and care trajectories of persons living with Hepatitis B in Senegal: A qualitative study
Source: PLOS Glob Public Health. 2025 Nov 7;5(11):e0005376. doi: 10.1371/journal.pgph.0005376 (PMC12594332; doi:10.1371/journal.pgph.0005376)
Supplement: Attachment — Submitted filename: SENB_CareTraj_reviewer_responses.docx [file pgph.0005376.s005.docx]

**Adrià Ramírez Mena, MD, MSc, PhD**

**Department of Infectious Diseases**

Inselspital, Bern University Hospital

CH-3010 Bern, Switzerland

adria.ramirez@posteo.net

**To the editors of *PLOS Global Public Health***

Dakar, August 6, 2025

**Response to reviewers of the manuscript “Experiences and Care Trajectories of Persons Living with Hepatitis B in Senegal : a qualitative study” (Manuscript ID PGPH-D-25-00055)**

Dear Editors,

Thank you for giving us the opportunity to respond to the reviewers and revise our manuscript. This document provides point-by-point responses to the reviewers’ comments, reproduced in *italics*. Changes to the **original text** are reproduced in **bold blue** (with corresponding line numbers of the document with track changes). We thank the reviewers for the time invested in reviewing our manuscript and their thoughtful comments, which helped improve our manuscript.

Yours sincerely,

Mariama Diedhiou, Albert Gautier Ndione and Adrià Ramírez Mena, on behalf of all co-authors

**Journal Requirements:**

*1. We note that you have indicated that there are restrictions to data sharing for this study. For studies involving human research participant data or other sensitive data, we encourage authors to share de-identified or anonymized data. However, when data cannot be publicly shared for ethical reasons, we allow authors to make their data sets available upon request. For information on unacceptable data access restrictions, please see* [*http://journals.plos.org/plosone/s/data-availability#loc-unacceptable-data-access-restrictions*](http://journals.plos.org/plosone/s/data-availability#loc-unacceptable-data-access-restrictions)*.*

We appreciate the editors’ comments and have noted the guidelines concerning data access restrictions. In line with these recommendations, we confirm that the data used from our study cannot be shared publicly due to its sensitivity, as it contains potentially identifiable information about participants. However, in accordance with PLOS policy, we will provide an anonymised dataset available upon reasonable request.

*a) If there are ethical or legal restrictions on sharing a de-identified data set, please explain them in detail (e.g., data contain potentially identifying or sensitive patient information, data are owned by a third-party organization, etc.) and who has imposed them (e.g., a Research Ethics Committee or Institutional Review Board, etc.). Please also provide contact information for a data access committee, ethics committee, or other institutional body to which data requests may be sent.*

We thank the editors for these clarifications regarding the data sharing policy. In accordance with your recommendations, we provide the following clarifications: the data in this study does indeed contain potentially identifiable information about participants (detailed demographic data, precise geographical location). These restrictions were imposed by the National Ethics Committee for Health Research No. 00000061 MSAS/CNERS/CP, which oversees this project. Requests for access to anonymised data can be sent to:

Name of data access manager : Babacar Thiendella Faye

Institution : Service des Maladies Infectieuses et Tropicales du Centre Hospitalier National Universitaire de Fann

Email: thiendella.faye@smit.sn

*b) If there are no restrictions, please upload the minimal anonymized data set necessary to replicate your study findings to a stable, public repository and provide us with the relevant URLs, DOIs, or accession numbers. Please see* [*http://www.bmj.com/content/340/bmj.c181.long*](http://www.bmj.com/content/340/bmj.c181.long) *for guidelines on how to de-identify and prepare clinical data for publication. For a list of recommended repositories, please see* [*https://journals.plos.org/plosone/s/recommended-repositories*](https://journals.plos.org/plosone/s/recommended-repositories)*. You also have the option of uploading the data as Supporting Information files, but we would recommend depositing data directly to a data repository if possible. Please update your Data Availability statement in the submission form accordingly.*

We thank the publishers for their clarification regarding data sharing.

We confirm that restrictions apply to our data due to its sensitive nature. In accordance with PLOS policy, we propose to make partially anonymised data available upon request. We have updated the statement regarding data availability in the submission form, as well as in the subsection ‘Availability of data and materials’ of the ‘Declaration’ section, (page 26) as follows:

**The data that support the findings of this study are available from ~~Albert Ndione Gautier~~ Babacar Thiendella Faye** **but restrictions apply to the availability of these data, which were used under license for the current study, and so are not publicly available. Data are however available from the authors upon reasonable request and with permission of Babacar Thiendella Faye ~~Albert Ndione Gautier.~~**

*2. We ask that a manuscript source file is provided at Revision. Please upload your manuscript file as a .doc, .docx, .rtf or .tex.*

We have uploaded the source file of the manuscript in .docx format as requested.

*3. We have noticed that you have uploaded Supporting Information files, but you have not included a list of legends. Please add a full list of legends for your Supporting Information files after the references list.*

We apologise for this oversight. We have inserted the list of legends after the references in the revised version of the manuscript (page 34)

**Comments to the Author: Reviewer #1**
*The manuscript is well written, and it's highly relevant given the WHO’s 2030 target for hepatitis elimination and the persistent challenges in HBV care in sub-Saharan Africa. The study contributes to individual lived experience in Senegal. It's well executed with clear relevance to public health*

We thank the reviewer for their positive evaluation and comments on our manuscript. We appreciate their attention to the significance of our study regarding the 2030 hepatitis elimination targets and the challenges of HBV management in sub-Saharan Africa.

**Comments to the Author: Reviewer #2**

*Congratulations on this worthy and interesting research, which includes data that has really interesting implications for public health responses to hepatitis B. I would like to make several suggestions to strengthen the manuscript:*

*1. The manuscript is generally well written, although there are several times when grammatical choices needed to be reviewed. I would suggest an independent edit of the manuscript and challenge assumptions made by the authors including where several sentences required a verb or where there was confusion about past and present tense.* *One minor issue in the abstract was noting that the aim of the manuscript was “identifying individual, social, and structural determinants influencing HBV care pathways thereby informing targeted interventions to improve HBV care delivery”, when the research was focussed on influencing access to care delivery. The use of HBV or hepatitis B needs to be consistent across the manuscript, and hepatitis B is not a hereditary disease.*

We thank the reviewer for their suggestions, which will improve our manuscript. We have addressed all comments and made the following corrections: a thorough linguistic review, systematic correction of grammatical errors and ambiguous wording, and standardized term usage: HBV is used consistently except in the first mention, and erroneous references to heredity have been removed. We have also adapted some sentences in the in “Abstract” section (page 2):

**“Hepatitis B virus (HBV) infection is a public health concern in Senegal, where individuals encounter multifaceted barriers in their care trajectories**.**This qualitative study aimed to identify the individual, social, and structural determinants influencing HBV care delivery to inform targeted interventions for improving ~~HBV care delivery~~ access. Between February and April 2021,we included 29 adults living with HBV~~,~~ from the SEN-B, a prospective HBV cohort in urban Senegal providing biannual clinical monitoring for HBV-related liver complications. We conducted individual semi-structured interviews, during which participants shared their experiences about care trajectories. Through inductive analysis, we identified four primary factors ~~were identified as~~ that shape care trajectories: diagnostic circumstances, disclosure choices, social environment, and financial burden.”.**

*2. I note that the journal does not have a word limit; this is one manuscript that would be strengthened by a word count*

We appreciate the reviewer’s suggestion to refine the manuscript’s conciseness. While the journal does not impose a strict word limit, we agree that tightening the text could improve clarity and focus. We have carefully revised the manuscript to streamline redundant sections, tighten phrasing, and ensure that all content contributes meaningfully to the study’s objectives.

*3. There are several times when sentences have been repeated with only a minor revision.*

Please refer to the reviewer answer to point 2.

*4. Sections of the introduction are better included within the discussion.*

We thank the reviewer for this suggestion. We believe that the relevant elements of the introduction play a fundamental role in: 1) establishing the conceptual framework of the study, 2) justifying the relevance of the research, and 3) clearly defining the general objectives. These elements would lose their structuring function if they were moved to the discussion section. We recognize the importance of maintaining a streamlined manuscript and have therefore implemented revisions to enhance clarity in the introductory section. Specifically, we have refined key explanatory sentences and supplemented them with additional references in the first three paragraphs (page 3). These modifications strengthen our theoretical positioning while preserving the introduction’s crucial role in structuring the paper’s narrative arc and framing our research contribution.

Paragraph 1:

**However, these challenges may be exacerbated by economic and structural constraints, including inadequate healthcare resources ~~and~~ , a lack of specific national guidelines for HBV management and the concentration of specialised services in urban areas**[**(5,6)**](https://www.zotero.org/google-docs/?broken=P9aCd1)**.(Sow et al. 2022)**

Paragraph 2:

**~~Since its establishment in 1999, Senegal's National Hepatitis Control Program (PNLH) has faced several informational constraints, notably a lack of well-coordinated and adequately funded public education and information dissemination efforts~~**[**~~(7,8)~~**](https://www.zotero.org/google-docs/?broken=0ybEZE)**~~. Despite the improvements in prevention strategies, limited awareness and access to treatment currently centralized and not fully subsidized continue to present challenges~~**[**~~(9,10)~~**](https://www.zotero.org/google-docs/?broken=LXzmLc)**~~.~~**

**.~~Managing chronic diseases such as HBV in sub saharan Africa, requires a comprehensive care pathway that goes beyond medical treatment. Significant social, economic, and cultural factors that influence individuals' decisions to seek care need to be addressed~~**[**~~(11,12)~~**](https://www.zotero.org/google-docs/?broken=tWulaN)**~~.~~ Beyond economic and structural barriers, HBV management in sub-Saharan Africa faces significant sociocultural challenges, including limited disease awareness among both populations and healthcare providers, along with persistent stigma (8,9). Despite progress in prevention strategies, awareness remains insufficient and access to treatment often centralized, continues to be limited (10,11). Effective management of HBV requires a holistic approach that integrating not only medical care but also the social, economic, and cultural determinants influencing healthcare access (12,13)**

**~~These factors play a crucial role in shaping individuals' experiences and therapeutic journeys, leading to the utilization of various treatment modalities such as traditional medicine, self-medication, and biomedicine~~**[**~~(13,14)~~**](https://www.zotero.org/google-docs/?broken=wC1I8z)**~~. This diversity, while reflective of individual representations, can hinder timely access to biomedical treatments, ultimately impacting public health outcomes, particularly in the management of chronic conditions~~**[**~~(15)~~**](https://www.zotero.org/google-docs/?broken=HSev3y)**~~.~~ In Senegal, as is the case for other chronic diseases such as diabetes and hypertension, economic constraints and limited access to standards of care often compel individuals to seek treatment only during severe illness or exacerbations**[**(16,17)**](https://www.zotero.org/google-docs/?broken=Ku67Mt)

Paragraph 3:

**To improve access to healthcare and enhance health outcomes, it is essential to examine care pathways with a focus on individual experiences. ~~The scientific literature~~ Current research on ~~the~~ HBV management ~~of HBV hepatitis B~~ in Senegal ~~is~~ remains fragmented, ~~although various aspects have been explored. Few~~ with studies ~~have comprehensively followed the entire course of persons living with hepatitis, from initial screening at long-term follow-up~~ addressing isolated aspects of the disease but lacking a comprehensive analysis of the full individual journey—from screening to long-term follow-up. ~~Additionally~~ Furthermore, ~~amplifying the voices of individuals living with HBV is crucial;, as their experiences and perspectives are often marginalized in discussions about healthcare delivery and policy decisions~~ while individual perspectives are critical for shaping effective health policies and services, the lived experiences of individuals with HBV remain underrepresented in existing literature** [**(16)**](https://www.zotero.org/google-docs/?OZCRJy)**. We aimed to address existing gaps in the understanding of care trajectories for individuals living with HBV in urban Senegal.**

*5. I was unable to check statements that had been referenced as I was unwilling to use Zotero.*

We apologize for the inconvenience caused by the Zotero-linked references in the initial submission. In the revised manuscript, all references have been formatted as standard citations in the journal’s required style, with no links to Zotero or other reference management software.

*6. I’m not sure framing the analysis of the data along care pathways – which should be fully described for readers – and whether or how this relates to the “mapping of care trajectories”. I would suggest that the hepatitis B cascade of care used by the WHO be used as a framing device for the results. This would give the authors the opportunity to reflect a commonly understood logical progression, and where barriers to the different aspects of the lived experience of people with hepatitis B in Senegal such as stigma, the use of traditional medicines and economic burden of health service access could be addressed.*

We appreciate the reviewer’s suggestion to use the WHO care cascade as an analytical framework. While this model is valuable in epidemiological studies for quantifying gaps in HBV management, its standardized stages may not fully capture the realities of care pathways in Senegal’s under-resourced health system. To conduct an in-depth analysis of the social, cultural and individual dynamics influencing the trajectories of people living with HBV, our study adopted a qualitative approach based on ‘Grounded Theory’ (Glaser & Strauss, 1967). This inductive methodology aims to construct a theory rooted in empirical data rather than applying a predefined analytical framework. Particularly suited to the Senegalese context, which is characterised by the coexistence of formal and informal healthcare systems, ‘Grounded Theory’ makes it possible to understand the non-linearity and discontinuities of trajectories, while grasping the rationales for action and the subjective meanings attributed to the disease.The analysis followed a rigorous process of open coding to identify emerging categories, then axial coding to establish links between these categories for example, between limited knowledge about HBV, stigmatisation, and interruptions in follow-up. This approach provided a detailed reading of the mechanisms underlying the trajectories observed.

*7. While recognising that African countries have a specific social, cultural and historical context in responding to hepatitis B, many of the issues raised in the results have been found in other countries, and which could be referenced.*

We have added references from other geographical contexts in the paragraph 3 “Discussion” section (page 25) as follows:

**Collectively, these findings indicate a pervasive and generalized lack of understanding of HBV among both the general population and healthcare providers, representing a significant barrier to accessing screening services. This observation is consistent with the findings from low-endemic countries such as the US, where similar gaps in understanding of the modes of transmission have been documented (Freeland et al. 2021).** **Participants in our study reported that the information provided during the initial announcement of their HBV diagnosis was often inadequate and difficult to understand.**

Paragraph 2 (page 25)

**Studies investigating barriers to HBV screening across African facilities have consistently reported low knowledge levels among healthcare professionals** [**(27–30)**](https://www.zotero.org/google-docs/?jaK6zL)**. These deficiencies extend more broadly to HBV awareness, as confirmed by studies conducted in various contexts, affecting both healthcare providers and the general population (Subic et Zoulim, 2018; Mursy et Mohamed, 2019). Collectively, these findings indicate a pervasive and generalized lack of understanding of HBVhepatitis B among both the general population and healthcare providers, representing a significant barrier to accessing screening services.**

Paragraph 5 (page 26)

**Our study revealed widespread self-stigmatization among participants, aligning with literature that identifies stigma as a significant barrier to disclosure and access to care** [**(35)**](https://www.zotero.org/google-docs/?DM5Ldb)**. Similar studies conducted both in HBV endemic and non-endemic regions have also documented stigmatizing attitudes, reflecting social avoidance, and thus contributing to social isolation and delayed access to care** [**(36,37)**](https://www.zotero.org/google-docs/?UFPGtV)

*8. The use of SEN-B to recruit participants is an interesting choice given the intrinsic bias of people with hepatitis B who enrolled into a hepatitis B study who would be more likely to be engaged in the health system and being more likely to have greater knowledge of the condition.*

We thank the reviewer for raising the important issue of potential selection bias. Our recruitment strategy for the SEN-B cohort was rigorously designed to enhance generalizability by systematically integrating multiple population-based sources: community screening campaigns, blood donor databases, and health facility referrals ( primary care and antenatal clinics), as previously published (Ramírez Mena et al., *The Lancet Gastroenterology & Hepatology*, 2024).

Regarding concerns about prior HBV knowledge as a confounder, we implemented a purposive sampling framework to ensure proportional representation across key strata: age, educational level, and healthcare access levels. Crucially, time since diagnosis was incorporated as an a priori stratification variable during sampling to prevent overrepresentation of long-term diagnosed individuals with greater health literacy.

9. The interview guide needs to be included

We have added the complete interview guide as a supplementary information.

10. More information is needed to rationalise why “participants were asked to express their concerns about the disease and its treatment.” and how this affected the research

We thank the reviewer for the opportunity to clarify our methodology. Eliciting participants' to voice their concerns about HBV and their treatment experiences was integral to our anthropological approach, as it illuminated the sociocultural dimensions of illness and healthcare navigation. This practice aligns with patient-centered care principles in public health research, ensuring that the research process itself became a space for reciprocal engagement, where participants’ lived realities could inform both empirical findings and clinical practice. The inclusion of patient concerns strengthened the study’s validity by grounding findings in participant-reported priorities rather than researcher assumptions.

Nonetheless, we have slightly adapted our text for more clarity in paragraph four, in “Data collection” sub-section of the “Material and methods” section as follows (page 7)

**“Verbal consent was recorded ~~before the start of~~ prior to each interview**. **~~At the start of each interview, p~~Participants were first asked to ~~express~~share their concerns about the disease and its treatment. The participants' concerns were incorporated into our initial interview protocol in order to capture the sociocultural dimensions of the disease and ground our findings in real-life experiences, in line with anthropological and person-centred care approaches. ~~At the end of each exchange, a professional health professional was available to answer their questions in a personalized manner~~ Following each interview, participants received individualized counseling from a study clinician to address their specific questions, with additional information sessions provided as needed.** **The face-to-face interviews were conducted in either Wolof or French, lasting between 45 to 90 minutes, and were held in a designated room at the Fann University Hospital of Dakar, Senegal”.**

*11. How was data saturation determined? The abstract only mentions inductive – how was deductive analysis used?*

Data saturation was defined as the point at which new interviews no longer contribute substantial information to the analytical categories (Saunders et al., 2018) and was assessed iteratively in our study. Following 26 interviews, the analysis indicated no emergence of new themes regarding therapeutic pathways, with five categories identified. To further validate this saturation, three additional interviews were conducted with participants who had diverse profiles in terms of age, gender, marital status, educational level, and occupation. These final interviews did not yield any new categories or variations of existing categories, thereby confirming saturation. The coding of the transcripts indicated that all main categories were supported by at least five subcategories and between 10 and 60 illustrative verbatim quotes, demonstrating the richness of the data.

We have now clarified this point in the “Data analysis” sub-section in the “Methods” section (page 8)

**“The data collection process continued until data saturation was achieved. The number of interviews required to reach saturation was determined iteratively ~~during~~ throughout the study, to ensure the completeness of the data collected, accompanied by ~~with~~ regular data analysis (Saunders et al., 2018)** [**(22)**](https://www.zotero.org/google-docs/?Lpg835)**. We ~~utilized~~ employed ~~both deductive and~~ inductive approaches~~.~~ with ~~C~~codes ~~were~~ initially defined based on the study's themes, and new emerging themes identified during the analysis of the interview transcripts. ~~from the interviews were identified during the reading process.”~~**

Furthermore, our research used an inductive approach based on ‘Grounded Theory’ to explore the dynamics of HBV care pathways in Senegal (see response to comment 6). This approach was essential for understanding the complexity of care pathways in a context marked by pluralism in healthcare systems, avoiding pre-established conceptual frameworks. Unlike a deductive approach that applies existing theoretical models, our method enabled the construction of knowledge directly from empirical data.

*12. The phrase “corresponding semester” needs clarification, as does the provision of consent which is described as both verbal and written.*

We thank the reviewer for the comment. Since participants often could not recall exact dates, the timing of each care interaction was captured and rounded to the nearest semester (e.g., first or second half of the year).

We have adapted the sentence in paragraph two of “Data analysis” sub-section in the “Material and Methods” section (page 8).

**“The resources used at each time point were categorized inductively by one researcher (ARM) and subsequently reviewed by two additional researchers (MD and AGN) to ensure consistency in the classification process. The ~~precise~~ timing of each event was recorded and rounded to the nearest semester (e.g., first or second half of the year)** **~~within the corresponding semester to enhance visual interpretation~~. A visual diagram was then developed to ~~illustrate~~ map each individual's ~~journey~~ trajectory through the healthcare system, displaying interactions at the semester level”.**

Written consent (signed form upon inclusion in the SEN-B cohort) and verbal consent (recorded pre-interview) comply with the requirements of the SEN-B cohort and our qualitative research. Clarifications regarding consent are provided in the “Ethical considerations” section (page 8).

**Ethical approval for the study was obtained from the National Ethics Committee for Health Research at the Ministry of Health and Social Action of Senegal (0061/MSAS/DPRS/CNERS). Written consent was obtained systematically via a signed form at the time of inclusion in the SEN-B cohort. ~~Each participant provided written informed consent consent, which.~~ It included the objectives, procedures, potential benefits, and risks associated with the study. Participants were made aware of their right to withdraw from the study at any time without needing to give a reason. Verbal consent was reaffirmed and recorded before each individual interview. It included a reminder of the specific objectives of the interview, explicit authorisation for audio recording and confirmation of understanding of the right to interrupt. ~~Consent was also requested for audio recording the discussions, and all participants consented to being recorded during the interviews~~ To ensure confidentiality, the recordings were conducted anonymously, with no use of participants' names**

*13. Does “Date of screening” in Table 1 actually mean date of diagnosis?*

We thank the reviewer for this observation. To clarify: The dates indicated in Table 1 correspond to the dates of diagnosis reported by the participants themselves during the interviews. We have changed the heading in table 1 from **~~Date of screening~~** to **Date of diagnosis** for greater clarity.

*14. Several quotes are used to illustrate a point but that do not relate to the introduction to the quote or the broader preceding paragraph, while a quote for women being diagnosed through antenatal consults would be useful.*

We appreciate the reviewer’s comment. All verbatim quotes have been reviewed for contextual accuracy. The verbatim included in “HBV diagnosis” sub-section of the “Results” section in paragraph 7 (page 13) have been repositioned to strengthen narrative coherence as follows:

**After participating in blood donation campaigns, some individuals reported not retrieving their test results due to distance to the healthcare facility and time constraints.**

**“I donated blood with my daughter. But she didn't pick up the results because she works as a housekeeper and doesn't have time to go". (Tida, ID #29)**

**As a result, often they only learned their HBV status after subsequent donations at different locations.**

**~~"I donated blood with my daughter. But she didn't pick up the results because she works as a housekeeper and doesn't have time to go". (Tida, ID #29)~~**

Changes have also been made in paragraph 13 (page 14)

**~~Typically, it was a healthcare professional who informed about the diagnosis, providing varying levels of explanations based on their own understanding and expertise.~~ This moment of revelation often occurred for individuals who had not initially intended to undergo screening.**

**~~“Looking at my health booklet, I noticed a cross marked in the hepatitis section and thought, "What the hell is this? I was so shocked that I dropped my bag on the bus. After that moment, I found it difficult to eat, sleep or do anything. I was so upset that I wore the same clothes for days -I just didn't feel like myself”. (Mame Ami, ID #13)~~**

We have also incorporated the following verbatim to add more clarity paragraph 11 (page 14)

**For women of reproductive age, antenatal consultations emerged as a common context for the unexpected revelation of HBV status.**

**“During my sixth pregnancy, I was asked to complete medical tests. When I returned with the results, I showed them to my midwife. She was the one who told me I had hepatitis B. After going home, I was so distressed that my blood pressure rose." - Mariama, ID~~#~~**

**Moreover, medical and paramedical students reported undergoing routine HBV screening before or during their internships, reflecting the integration of HBV awareness into their educational or professional experiences.**

*15. Assumptions are made throughout the manuscript of knowledge of the Senegal health system. One example is that it is unclear why a person would need to be “retrieving their test results”.*

The failure to retrieve post-donation results can be explained mainly by three factors: the initial motivation of donors, which focuses on the act of generosity rather than screening; a lack of information about the usefulness of retrieving results, despite the systematic analysis of each donation; and geographical and financial constraints related to access to the National Blood Transfusion Centre, which is often located far from people's residences. These factors are exacerbated by the lack of awareness campaigns and limited access to reliable information, as previously shown by Ndione. AG, et al (BMJ Open 2025).

The Senegalese healthcare system, marked by pronounced geographical and socio-economic disparities, presents major challenges in terms of access to care, particularly for chronic diseases such as hepatitis B. This situation is exacerbated by the concentration of specialised health facilities in Dakar, which forces patients from outlying regions to make often costly and dissuasive journeys. Unlike digitised healthcare systems where medical results are automatically sent to individuals, the Senegalese model still relies heavily on physical collection of results. As a result, donors must frequently travel to the National Blood Transfusion Centre (CNTS), often located dozens of kilometres from their homes, to obtain their results, creating a logistical and financial barrier, particularly for low-income populations.

*16. The following paragraph included in page 13 needs clarification – it sounds really interesting but is very unclear in its meaning: Those who chose to share their positive status … through media sources, particularly online platforms.*

We thank the reviewer for this observation. The paragraph has been restructured for clarity. We have provided clarifications regarding consent in paragraph six of the “Choices about HBV disclosure” subsection in the “Results” section (page 16 - 17).

**~~Distinct pathways emerged based on participants’ choices to disclosure. Those who chose to share their positive status typically engaged in discussions about care options with close relatives, and occasionally consulted medical practitioners. In contrast, individuals who did not receive support or advice from those closest to them, especially in the absence of family members working in the healthcare field, frequently sought information about the disease through media sources, particularly online platforms.~~ The analysis reveals two distinct information management strategies: participants with a supportive social network generally shared their status and accessed care more easily through family advice. Others, particularly those without relatives in the medical field, developed informational autonomy through online media.**

*17. Linkage to care requires a section in and of itself, and the section describing the role of social environment on therapeutic journeys could be more clearly and accurately titled.*

We recognise the value of a section dedicated to linkage to care, but believe that our cross-cutting approach, which integrates these aspects throughout the article, offers greater analytical consistency. This integration avoids redundancy while preserving the complexity of the interactions between the various factors influencing healthcare trajectories. We kindly refer to our response to comment 6

We have adapted the title of the above referred sub-section (page 17)

**~~The role of social environment on therapeutic journeys~~** **Role of social and community networks in therapeutic choices**

*18. Many of the findings included in the discussion have been found in other settings and could be referred to, particularly in the non-clinical aspects of living with hepatitis B.*

Here we kindly refer to our response to comment 7 of reviewer 2.

*19. Issues such as “medical wandering” could be better defined, with self-stigmatisation not being widely described in the results, while the relationships between therapeutic pluralism and sociocultural practices is unclear.*

We thank the reviewer for these comments, which have helped to improve the accuracy of our manuscript.

Medical wandering refers to the phenomenon where patients or their families navigate through multiple healthcare providers, facilities, or services — often in an uncoordinated or inefficient manner — while seeking diagnosis, treatment, or adequate care. We have adapted paragraph two and three of the “Discussion” section for better clarity (page 25).

**“Participants in our study reported that the information provided during the initial announcement of their HBV diagnosis was often inadequate and difficult to understand. The perceived technicality of status announcements generated confusion, eroding trust in healthcare providers and prolonging care-seeking delays. This contributed to protracted intervals between diagnosis disclosure and formal healthcare engagement, exemplifying patterns of medical wandering—a phenomenon previously documented in sub-Saharan Africa (36). Such trajectories were shaped by intersecting structural vulnerabilities, psychosocial barriers, and cultural interpretations of illness, underlining the need for contextually adapted chronic care models”**

To enhance the conceptual clarity and empirical grounding of self-stigmatization within our analysis, we have refined the explanatory text and incorporated a supporting verbatim quotation in paragraph nine of the "Choices about HBV Disclosure" subsection (Results section, page 14).

**“A significant barrier to disclosure was the insufficient information regarding transmission risks, clinical manifestations, and the progression of the disease and self stigmatisation.**

**This ignorance fuelled self-stigmatisation, often resulting in social self-exclusion. Some participants adopted excessive behaviours for fear of infecting their loved ones.**

**“I don't go anywhere anymore, I isolate myself [...]. I also have my own spoon because I don't want to risk infecting my family” (Mame Ami, ID #13)**

**This quote illustrates how the fear of transmitting HBV, although medically unfounded, can lead to isolation strategies and excessive precautions.This fear and uncertainty frequently resulted in hesitance to share their status, which not only affected the dynamics of family screening practices but also contributed to the trivialization or denial of the illness”.**

Furthermore, the links between therapeutic pluralism and sociocultural practices center on key mechanisms in our study. Therapeutic orientation is strongly influenced by social networks, particularly family networks. Economic and accessibility considerations also come into play, as participants often perceive traditional medicine and natural remedies as cheaper and more accessible, thereby favouring fragmented care pathways.

20. *This research is important and its findings are necessary for the development of an effective public health response to eliminate hepatitis B as a public health threat.*

We thank the reviewer for his appreciation of the importance of our research in the context of HBV elimination. We fully share this vision and emphasise that our study is part of a public health response adapted to the Senegalese context. The data collected on therapeutic trajectories, social representations and shortcomings in the healthcare system offer actionable avenues for: improving screening strategies (particularly at key periods, strengthening post-diagnosis psychosocial support …)
